# Getting back on track: Development of a blended-care intervention for weight recurrence after metabolic bariatric surgery using intervention mapping

**DOI:** 10.1016/j.pecinn.2025.100404

**Published:** 2025-05-18

**Authors:** Vera Voorwinde, Ingrid H.M. Steenhuis, Ignace M.C. Janssen, Valerie M. Monpellier, Maartje M. van Stralen

**Affiliations:** a Dutch Obesity Clinic, Huis ter Heide, the Netherlands; b VU University, Faculty of Science, Department of Health Sciences and Amsterdam Public Health Research Institute, Amsterdam, the Netherlands

**Keywords:** Metabolic bariatric surgery, Lifestyle intervention, Intervention mapping, Weight recurrence, eHealth, Patient participation

## Abstract

**Objective:**

To systematically re-develop a blended-care intervention addressing weight recurrence after metabolic bariatric surgery (MBS). Weight recurrence poses a significant longterm challenge for around 20–30 % of MBS patients. The intervention aims to improve weight outcomes and enhance patient well-being. This study describes the novel application of the Intervention Mapping (IM) protocol, integrating scientific evidence and stakeholder input.

**Methods:**

The six-step IM protocol guided the development process, ensuring the active involvement of patients and healthcare professionals. A comprehensive needs assessment using quantitative, qualitative, and literature-based approaches informed the creation of a logic model of the problem (Step 1) and a logic model of change (Step 2). Program outcomes and objectives were formulated through collaborative brainstorming and design-thinking sessions, leading to intervention design (Step 3). The intervention was co-produced with patients, implementers, and an app developer (Step 4). Detailed implementation (Step 5) and evaluation (Step 6) plans were subsequently developed.

**Results:**

The IM process yielded a blended-care intervention grounded in theoretical frameworks and evidence-based methods. The intervention actively involved the target population and implementers, addressing key determinants of weight recurrence.

**Conclusion:**

The IM protocol demonstrated utility in designing a tailored, theory-based intervention post-MBS. The process emphasized the value of integrating stakeholder perspectives and highlighted the feasibility of co-creating an evidence-informed intervention.

**Innovation:**

This intervention incorporates newly developed elements in a novel blended-care structure. Future evaluation is necessary to determine its effectiveness in achieving the desired outcomes.

## Highlights

• Intervention Mapping facilitated the active involvement of both patients and implementers.
• Patients attribute weight recurrence to life events and stressors, or a lack of social support.
• The intervention focuses on stress management, healthy eating, planning, and strategies for relapse prevention.
• Personalized modules and goal-setting tools were integrated into the intervention using a blended-care approach.
• Professionals emphasized self-direction, autonomy and skill-building as crucial elements.

## 1 Introduction

Obesity is a complex, chronic disease affecting over 1 billion people globally as of 2022 [1]. Treatment typically depends on a person's body mass index (BMI), with metabolic bariatric surgery (MBS) being the most effective option for people living with severe obesity (BMI ≥ 40 or ≥ 35 with associated medical problems) [[2], [3], [4]]. MBS is associated with reduced overall mortality, increased life expectancy, and sustained health improvements, such as type 2 diabetes (T2DM) resolution, along with a mean weight loss of approximately 30 % of total body weight within 1.5 years post-surgery [2,3,[5], [6], [7], [8]]. However, approximately 20–30 % of patients experience weight recurrence, presenting a significant long-term challenge [[9], [10], [11], [12], [13], [14]]. This weight recurrence is recognized to increase the risk of diverse adverse health outcomes, including deterioration of T2DM, dyslipidemia, psychosocial distress, and Health Related Quality of Life (HRQoL), exhibiting a dose-response correlation [9,11,[14], [15], [16], [17], [18]].

Weight recurrence is believed to have a multifactorial cause, encompassing genetic, endocrinological, anatomical, behavioural, and psychological factors [[19], [20], [21], [22], [23]]. Therefore, a multidisciplinary approach to addressing weight recurrence is generally recommended, with lifestyle interventions serving as the cornerstone and, in most cases, the initial step in interventions to prevent or reverse weight recurrence [[24], [25], [26], [27], [28]]. However, while multidisciplinary lifestyle interventions have been implemented in BMS care, there are no established guidelines regarding such interventions.

Studies on interventions addressing weight recurrence are very scarce [24,28]. The few studies that do describe (guidelines for) lifestyle interventions involve behaviour modification or psychological treatments and focus on nutritional guidance, self-monitoring of food intake and physical activity, and the development of coping skills to manage emotional eating [19,24,29,30]. The Back on Track (BOT) intervention, developed by the Dutch Obesity clinic (Nederlandse Obesitas Kliniek, NOK) is such a concise lifestyle intervention based on motivational interviewing to promote a healthy lifestyle and weight stabilization [31]. The current BOT consists of 3 in-person consultations with an HCP (psychologist, dietitian, physiotherapist) with 4 weeks between each session, followed by an evaluation with the physician 2 months later. The intervention aims to stabilize weight by addressing all relevant lifestyle factors. However, the intervention has not yielded the desired outcomes in terms of weight stabilization, patient engagement and retention [31]. Similar issues are observed in other long-term care programs, where attendance rates tend to be consistently low [[32], [33], [34]].

A potential reason for these challenges is that patients and HCPs are often not systematically included in the development process of the programs. By incorporating patient and HCP participation the adoption, implementation, engagement, and retention could be enhanced, and consequently the effectiveness of the program could be improved [35]. Another reason for the lack of intervention effectiveness could be the lack of systematic development of the interventions. Interventions developed based on theory and scientific evidence, often yield superior results [[36], [37], [38]]. Of the limited interventions specifically targeting the management of weight recurrence after BMS, only a few are grounded in theory and scientific evidence [30,39]. Intervention Mapping (IM) provides a structured approach for the development and implementation of effective health promotion interventions tailored to the needs of the target population and implementers [37].

Previously, a comprehensive needs assessment was conducted to assess perspectives and experiences of patients and HCPs with the current BOT program [31]. This assessment identified several areas for improvement including: enhancing accessibility of the intervention, integration of the intervention within standard care to mitigate feelings of shame and exceptionalism and, tailoring the intervention to meet individual patient needs through a comprehensive assessment of factors influencing weight recurrence (including lifestyle imbalances and stress) [31]. eHealth was identified as a promising complement to the existing intervention, offering opportunities for increased personalization, early screening, enhanced patient autonomy, and improved accessibility [31].

The objective was therefore to develop a blended-care intervention, defined as an integrative approach that combines face-to-face treatment with digital eHealth tools. This blended approach was built upon the existing in-person BOT intervention. However, the new intervention, referred to as the BOT 2.0, represents a substantial re-development, involving the creation of new content, the integration of newly developed eHealth components, and the transformation of the intervention into a blended-care format.

In this paper, we describe the novel application of Intervention Mapping to the re-development of the BOT into a blended-care intervention for addressing weight recurrence following MBS.

## 2 Materials and methods

### 2.1 Setting

The BOT intervention is part of a perioperative program that lasts from 6 weeks before MBS to 5 years afterward [13]. The program primarily consists of group-based sessions designed to promote the development of a healthy lifestyle and to support patients in adapting to changes following BMS. An intensive phase is conducted over a 6-week period, starting prior to surgery and continuing up to one year postoperatively. The multidisciplinary treatment team comprises a dietitian, psychologist, physiotherapist, and physician. After completion of the intensive phase, a structured follow-up program is provided for up to five years post-surgery. The follow-up program consists of medical consultations and a physical test at 18,24,36,48 and 60 months after surgery. In addition, psychologists and dieticians are available for consultation if deemed necessary [31]. During the follow-up period, the BOT intervention is available. Patients are eligible for the BOT if they experience weight recurrence and/or a relapse in behaviours that may contribute to weight recurrence.

### 2.2 Intervention mapping

IM provides a structured approach that ensures that the intervention is well tailored to the needs of the target group, is based on scientific evidence and can be evaluated to assess its effectiveness [37]. An underpinning of IM, is the social ecological model, in which health is a function of individuals and their environment [37]. This approach aligns with our understanding of obesity as a complex, chronic disease with various biological, psychological, behavioural, and environmental factors that cause it and interact with each other.

**Fig. 1:**
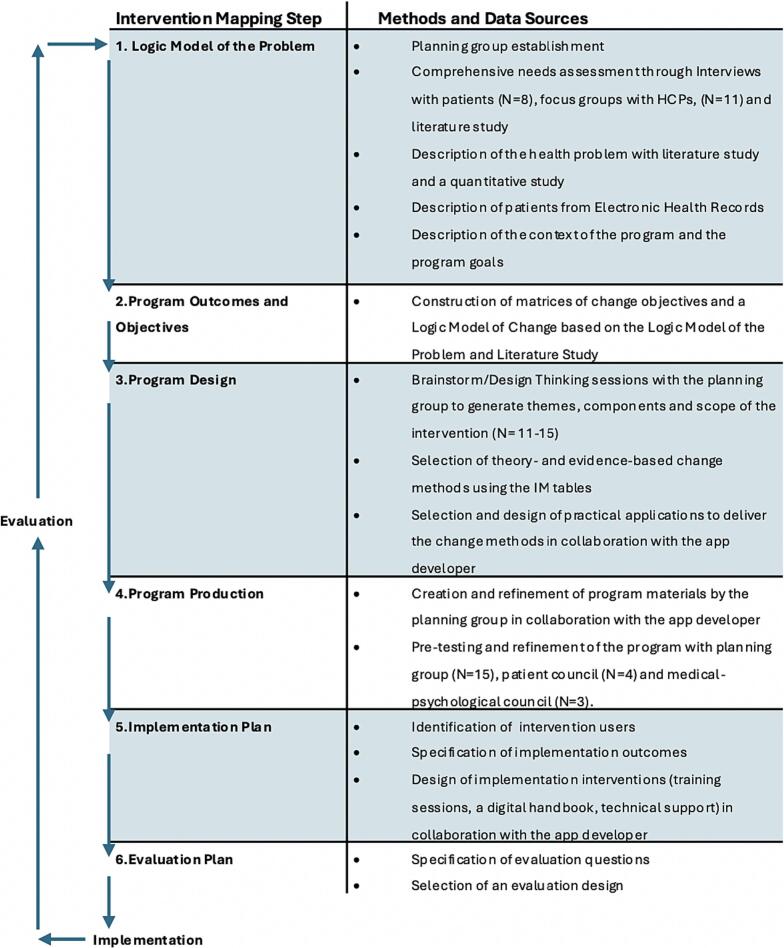
Methods and Data Sources for each Intervention Mapping step.

The IM Protocol consists of six steps that were all applied to the systematic development of the BOT 2.0. In the sections below we will describe how we have applied these steps. Fig. 1 schematically describes the development process.

#### 2.2.1 Step 1: logic model of the problem

In March 2019 we established a planning group comprised of staff members from the NOK, physicians, dieticians, psychologists, and physiotherapists, scientific experts in the field of health promotion, systematic intervention development, and weight loss maintenance, and organized the first meeting. During the development process the planning group was supplemented with intervention developers from a mental health app development company. Additionally, a patient advisory group consisting of 4 patients of the NOK were involved in the development process.

#### 2.2.2 Needs assessment

Next, from March 2019 until January 2020 multiple methods were employed to gather information for an extensive needs assessment. These methods included a literature review, 8 patient interviews with individuals who participated in the BOT, 3 focus groups involving a total of 11 HCPs associated with the BOT intervention, as well as meetings and brainstorming sessions with the planning group. A detailed description of how these data were collected and analysed, including the results can be found in a previous publication [31].

#### 2.2.3 Logic model of the problem

The initial step results in a Logic Model of the Problem, which schematically depicts the health problem, in this case weight recurrence after BMS, with the underlying behavioural and environmental causes. In addition, it depicts the determinants of these behavioural and environmental causes. We described the health problem, its impact on quality of life and the target group with literature review and a quantitative study [14].

#### 2.2.4 Step 2: Program outcomes and objectives, a logic model of change

The second step was to develop a logic model for the intervention in which the behavioural and environmental goals for the intervention were formulated and subdivided into performance objectives that specify the exact changes that will be addressed by the intervention to reach the program goals. First, the research team translated the behavioural factors from the needs assessment into desired health-promoting behaviours. These health-promoting behaviours were then subdivided into performance objectives. The third task involved selecting important and changeable determinants influencing health behaviours based on theoretical models from the literature.

Next, the Matrices of change objectives combine these personal determinants with performance objectives, creating specific goals that aim to modify the determinants that are crucial for effecting the desired behaviour change [37]*.* From the matrices of change objectives, a logic model of change can be constructed, which visualises the causal relationship between the determinants, the change in those determinants through the intervention, and ultimately the desired behaviour change.

#### 2.2.5 Step 3: Program design

In step 3 the intervention was shaped in terms of themes, components, scope, and sequence. Theory and evidence-based methods were selected and translated into practical applications to facilitate behaviour change**.** From March 2019 until the end of 2020, multiple brainstorming and design thinking sessions with the planning group, along with interviews with patients and focus groups with healthcare professionals, were conducted to generate ideas for the overall program. After the first design thinking session a prototype was made by the research team and extensively presented to both patients and healthcare professionals for evaluation and input. The resulting program outline served as the basis for selecting theory- and evidence-based change methods by the research team. In November 2020 collaboration with an app developer started, after which methods were translated into practical applications in collaboration with the app development company.

#### 2.2.6 Step 4: program production

Step 4 involved co-creating and pretesting all materials and protocols and making any necessary adjustments. The tables outlining different theoretical methods for change and corresponding practical applications from step 3 served as the foundation for development of the content**.** From November 2020 until November 2021 all materials were co-created, tested, and revised with HCPs from the relevant disciplines and the app developer. During the testing phase, the entire planning group and the client council extensively reviewed and tested the app.

#### 2.2.7 Step 5: implementation plan

Step 5 involved the development of an implementation plan. A plan was made that considers the practical aspects of implementing the intervention, such as training intervention users, setting a timetable and identifying the resources needed.

#### 2.2.8 Step 6: evaluation plan

Step 6 focuses on creating an evaluation plan. The evaluation will be described in more detail in a subsequent paper.

## 3 Results

Below we describe the main findings of each IM step.

### 3.1 Step 1: Needs assessment and logic model of the problem

#### 3.1.1 Logic model of the problem

**Fig. 2:**
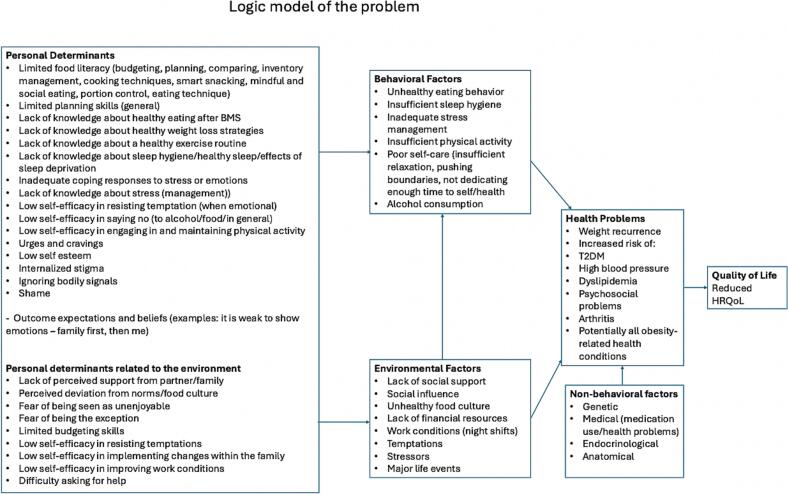
Logic Model of the Problem.

Fig. 2 shows the Logic Model of the Problem that was created based on the needs assessment. Patients from the NOK who experienced weight recurrence were identified as the target population. Among patients participating in the BOT, 80 % were women, with an average age of 47 (SD: 10.4) years, which is comparable to the entire population at the clinic [11]. On average, patients joined the program 3.9 years after surgery, with an average BMI of 35,4 kg/m^2^ (SD: 5.4).

Next, the literature review indicated that also after MBS weight recurrence, is a complex issue influenced by behavioural, psychological, physiological, and environmental factors. Examples of behavioural and psychological factors identified included unhealthy eating behaviours, inadequate stress management, and insufficient physical activity [[19], [20], [21], [22], [23],^40^,^41^]. The interviews provided further insights into the underlying causes of weight recurrence, confirming the view that weight recurrence is a multifaceted problem with diverse causes. Patients often attributed weight recurrence to psychological factors or environmental factors such as major life events and stressors, or a lack of social support [31].

Subsequently, personal determinants of the identified psychological, behavioural, and environmental factors were derived from the literature and from theories including social cognitive theory, theory of planned behaviour, and self-determination theory [[41], [42], [43], [44], [45], [46], [47], [48]]. Self-Determination Theory (SDT) distinguishes between autonomous and controlled forms of motivation. It posits that the fulfillment of three basic psychological needs—autonomy, competence, and relatedness—fosters autonomous motivation, which is more likely to support sustained health behaviour change. Evidence consistently indicates that autonomy support and perceived competence are critical factors in promoting long-term adherence to health-related behaviours [49]. Social Cognitive Theory (SCT) emphasizes the dynamic interaction between personal factors, behaviour, and the social environment. Key constructs include observational learning, reinforcement, and particularly self-efficacy, which has been consistently identified as a determinant of successful weight loss maintenance in several systematic reviews [41,50]. The Theory of Planned Behaviour (TPB) suggests that behavioural intention is the most important predictor of action, shaped by attitudes, perceived social norms, and perceived behavioural control [47,51]. While it is acknowledged that intention does not always translate into behaviour, it remains a relevant construct and was therefore included. Examples of these determinants included lack of skills, self-efficacy, knowledge, and experienced stigma.

#### 3.1.2 Program goals

The goal of the new BOT intervention (BOT 2.0) is similar to the previous intervention: to improve and/or sustain the positive effects of MBS in the long term, including achieving weight maintenance, improvement/stabilization of obesity-related medical problems, and enhancement of HRQOL. This objective is to be achieved by restoring patients' ability to maintain a healthy lifestyle.

### 3.2 Step 2: Program outcomes and objectives

Because it was not possible for the planning group to design an intervention that encompasses all factors influencing an individual's weight, the planning group prioritized and made a deliberate decision, guided by the outcomes of the needs assessment, to focus on four key outcomes: stress, healthy eating, planning, and relapse management. These behavioural outcomes were translated in specific performance objectives as depicted in Table 1.

**Table 1:** Behavioural outcomes and Performance Objectives.

Behavioural Outcomes (BO)	Associated Performance Objectives (PO)
***Patients will:***	***Patients will….***
BO.1. Manage stress in a healthy way	Utilize effective coping strategies when experiencing stress Make a conscious decision to prioritize relaxation Plan moments for relaxation Make the intentional choice to take time off for self-care Establish and maintain personal boundaries Communicate boundaries and individual needs effectively
BO.2. Follow nutritional guidelines for healthy eating after BMS	Make a deliberate decision to have six small meals throughout the day Consistently maintain the habit of consuming six small meals daily Choose to separate eating and drinking Continuously adhere to the practice of separating eating and drinking Monitor personal food intake regularly Evaluate dietary behaviours in relation to the recommended nutritional guidelines Develop a plan and take necessary actions to improve dietary behaviours Limit the consumption of unhealthy snacks Make conscious and mindful choices when selecting snacks Allocate sufficient time for meals Create a pleasant eating environment Employ appropriate eating techniques, such as chewing slowly and using smaller utensils Practice portion control by selecting a healthy serving size
BO.3. Plan meals and important activities	Plan meals in accordance with the nutritional guidelines post-BMS Plan important activities, including sports, relaxation, and social interactions Adhere to the planned schedule
BO.4. prevent relapse or get back on track after relapse.	Recognize high-risk situations that may lead to relapse Implement effective coping strategies when faced with high-risk situations Employ suitable coping mechanisms in the event of a relapse Monitor physical activity levels Monitor dietary behaviours Monitor personal weight

In the creation of performance objectives, we took a self-regulatory approach to support autonomy for the patients. For example, for the behavioural outcome that patients will follow nutritional guidelines for healthy eating after BMS, this behavioural outcome was broken down into self-regulatory performance objectives such as ‘patients monitor their own food intake’, and ‘patients evaluate their eating behaviour in relation to the nutritional guidelines’. At the same time, leaving room to choose the behaviours they want to change or, for instance, the type of eating behaviour they wish to address.

#### 3.2.1 Matrices of change objectives

Next, the matrices of change objectives were constructed by combining performance objectives with personal determinants. Examples of these objectives encompass identifying personal high-risk situations for relapse and demonstrating the ability to select appropriate coping strategies within such risky contexts. An excerpt from a change objectives matrix is depicted in Table 2.

**Table 2:** Matrix of Change Objectives.

[STRESS] Performance Objective:	Determinants					
	Knowledge	Skills	Self-efficacy	Normative beliefs	Outcome expectations	Percieved barriers
Utilize effective coping strategies when experiencing stress	K.1 Describe wat stress is K.2 State physical and emotional signs of stress K.3 List personal stressors K.4. List effective coping strategies	S.1. Demonstrate the ability to recognize one's own stress signals S.2. Demonstrate the ability to select adequate coping strategies	SE.1. Express confidence in ability to cope with stress	N.1. Recognize that significant others will understand if you need to express your emotions, or take time for yourself	OE.1. State that stress can be relieved by deploying adequate coping strategies OE.1. List the negative consequence of not dealing with stress adequately	PB.1. Anticipate the tendency to ignore stress
Make the intentional choice to take time off for self-care	K.1. State that a good balance between shoulds and wants is important for lifestyle balance and therefore works protective of relapse	S.1. Demonstrate the ability to prioritize themselves	SE.1 Express confidence in the ability to prioritize themselves	NB.1.1 State that significant others approve that I plan time for myself	OE.1.1 State that planning time for yourself will help in maintaining a healthy lifestyle	PB.1.1 Anticipate others asking for your time
Establish and maintain personal boundaries	K.1. List their boundaries within their relationships	S.1. Recognize their boundaries S.2. Demonstrate assertiveness S.3. Demonstrate the ability to say no	SE.1.1 Express confidence in setting healthy boundaries		OE.1.1 State that setting bounderies is healthy and contributes to maintaining a healthy lifestyle	PB.1. Anticipate others will not always accept your boundary

#### 3.2.2 Logic model of change

**Fig. 3:**
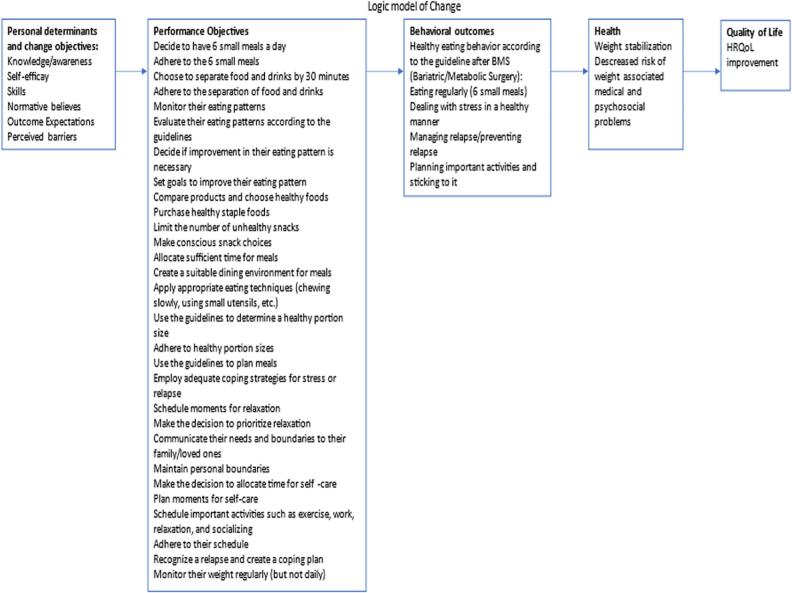
Logic Model of Change.

Figure 3 shows the logic model of change that was constructed.

### 3.3 Step 3: Theory based methods and practical applications

In the first task, the generation of ideas requires creativity and ‘thinking outside the box’ which is why we organized two design thinking sessions with the whole planning group, supplemented with marketing experts, to generate a diversity of creative ideas without real-world constraints [52]. The first session, held in March 2019, resulted in several ideas for the blended-care BOT 2.0. Among the key themes that emerged were the use of a visual communication tool, and a strong emphasis on self-direction, autonomy, and skill-building. Utilizing the visual communication tool for a “BOT check” emerged as an idea to facilitate the identification of specific factors to be targeted.

Following this session, the research team developed a prototype of the visual tool, BOT check, and an app module. Within the design thinking approach, prototyping plays a critical role in translating abstract ideas into tangible representations that can be iteratively refined through user feedback. This prototype was extensively presented to both patients and healthcare professionals for evaluation and input.

Based on the insights gathered, a second design thinking session was conducted in January 2020. During this session, the initial concepts were further refined and translated into more concrete intervention components, with a stronger focus on usability, clarity, and integration within the existing treatment structure.

Next, to delineate the patient journey, the insights from the design thinking sessions were integrated by the research team, aligning with the clinic scheduling, relevant literature and needs assessment. For instance, considering the Self-Determination Theory, the research team considered patient self-direction and autonomy as crucial elements [43,49]. To incorporate these aspects, we aimed to provide patients with the opportunity to choose which factor to focus on, coupled with relevant online modules. Online modules were then tailored to address each factor, emphasizing skill development. Recognizing the efficacy of more frequent contact, interim personal feedback moments were added [53]. Furthermore, patient interviews underscored feelings of isolation among those experiencing weight recurrence, prompting a desire for peer support. However, clinic constraints prevented the inclusion of such support within the program. Instead, a welcome module was incorporated into the intervention to normalize weight recurrence and elucidate its underlying causes, thereby facilitating patient engagement.

Next, theory-based methods for behaviour change were selected. Drawing upon the existing literature concerning eHealth interventions aimed at promoting healthy lifestyles, several important theoretical methods were identified, including monitoring, personalized mapping of target behaviours, and the utilization of goal setting and evaluation strategies [38]. Moreover, the planning group chose tailoring as an important method because patients expressed a desire for tailored modules to their specific needs [31]. Recognizing the influential role of health- and food literacy in shaping health behaviours, the planning group emphasized a focus on methods for skill-building within the intervention [42,45].

The last task in step 3 was to select practical applications. The planning group collaborated closely with the app development team to translate theoretical methods into practical applications. For example, an evidence-based method for skill building and self-efficacy, that is applicable to relapse situations according to attribution theory and relapse prevention theory is ‘Planning coping responses’ [37]. The practical applications are to list potential barriers in an entry exercise in the app and to subsequently fill in implementation intentions with ways to overcome these barriers. Table 3 shows an extract of a table with methods and applications. The complete tables can be found in appendix A.

**Table 3:** Theory based methods and applications.

Behavioural outcome:	Change objectives and determinants	Method	Application
Prevent relapse or get back on track after relapse.	Knowledge about lapse/relapse, that it is normal when changing behaviours, and doesn't have to be a problem when one manages to quickly get Back on Track	Information Framing	Information normalizing lapse/relapse
	Skills Recognizing relapse	Elaboration	Tick boxes and fill in personal signals of relapse
	Self-efficacy	Modeling	Peer video showing a coping model who got back on track
	Coping skills	Guided practice Planning coping responses	‘Traffic light’ practice in which high-risk situations, signals, and coping responses are filled in. Examples are filled in to guide patients
	Self efficacy	Providing reward Reinforcement	Milestone, motivational text Personal feedback
	Outcome expectations of monitoring	Information	Information about the importance of monitoring behaviours in relapse prevention
	Skills Monitoring	Guided practice Self-monitoring of behaviour	Example diary Activate in-app food diary Activate graph to monitor weight

### 3.4 Step 4: Producing Programme components and materials

**Fig. 4:**
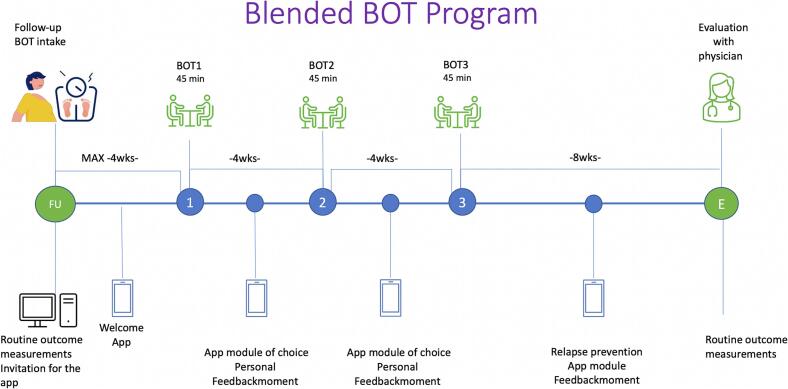
Patient Journey Back on Track (BOT) 2.0 intervention.

Based on previous research the decision was made to blend face to face contact with online contact and a mobile application (app) that offers various modules [31]. For the app, the interviews and focus groups underscored a preference for concise textual content, accompanied by audio-visual elements such as films and animations. During the testing phase, the entire planning group and the client council extensively reviewed and tested the app. In response to the feedback, the app was made more interactive. This involved increasing the use of methods for tracking goals and incorporating feedback mechanisms, such as providing results from a questionnaire suggesting following a specific module. Additionally, adjustments were made to the text to align with the treatment program and ensure an appropriate language level. Some feedback could not be implemented due to limitations within the app platform being utilized. For instance, the app maintained a rigid structure where completion of one section was necessary to progress to the next, whereas the desire was to enable revisiting and re-entering information. Furthermore, there was no provision for automated personalized feedback. Fig. 4 illustrates the flow (patient journey) for the blended intervention.

### 3.5 Materials

Below is a brief description of all the materials we have developed:

#### 3.5.1 Visual communication tool

**Fig. 5:**
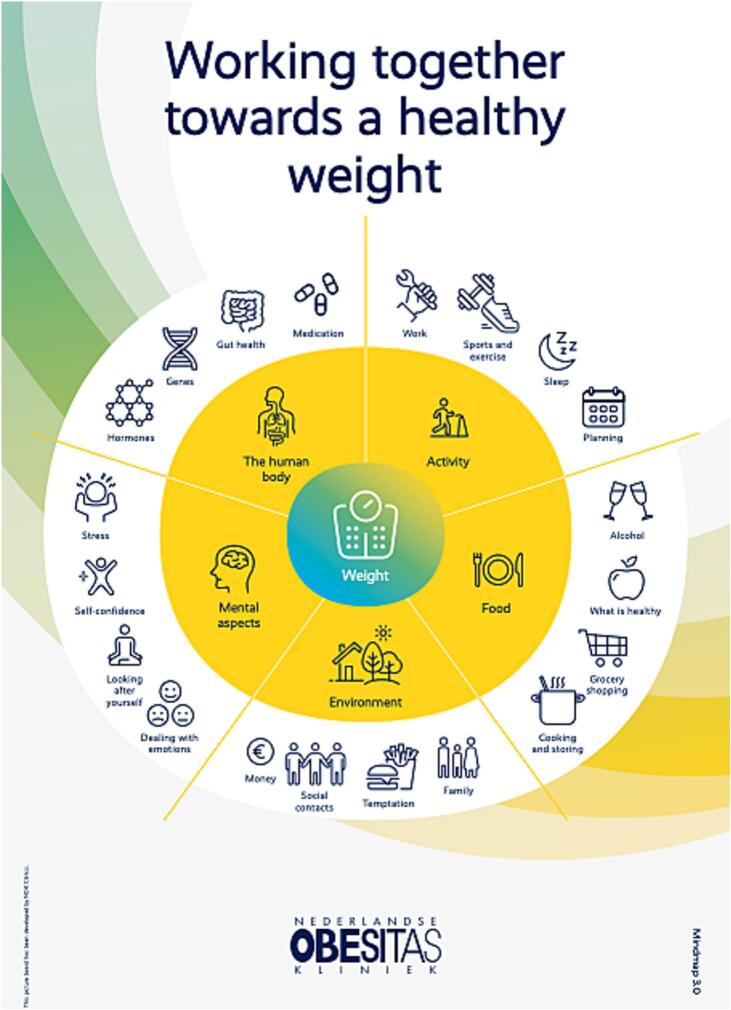
Visual Communication tool.

To make the logic model understandable and accessible to patients, a visual communication tool has been developed. This figure, in a simplified and visual manner, demonstrates all major factors influencing weight. We explicitly chose to also incorporate factors that cannot be changed, to show patients that the cause of obesity is multifactorial. The behavioural factors are subdivided into ‘activity’ and ‘nutrition’, alongside the environmental, psychological, and medical factors as described above. The tool is depicted in Fig. 5. It was made prominently visible throughout the offline and online treatment program, both in the consultation rooms and within the app.

#### 3.5.2 Welcome module

**Fig. 6:**
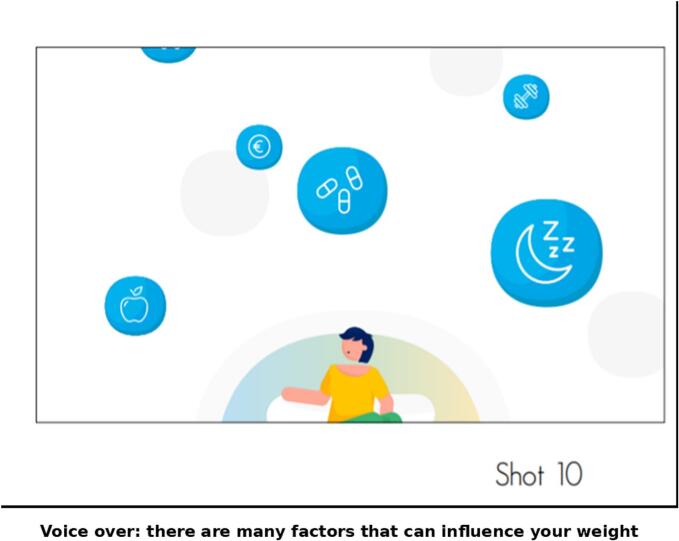
Screenshot from the welcome animation.

The welcome module begins with an animation that normalizes weight recurrence, explains the factors influencing weight recurrence, and outlines how these factors will be addressed in the BOT. The client council was involved in creating an engaging character as the main character in the animation and ensuring its comprehensibility (See Fig. 6).

#### 3.5.3 Back on track questionnaire

The welcome module automatically prompts a screening questionnaire that covers all areas of concern. This questionnaire provides results in colours: green, orange, red, indicating whether something is going well (green), deserves attention (red), or offers room for improvement (orange). This allows for awareness in a clear overview of the areas that require attention in an easily understandable manner. It also incorporates a level of tailoring as the results provide recommendations for the modules to follow.

**Videos featuring individuals with lived experiences**: To address patients' feelings of being the only ones experiencing weight recurrence, a male and a female were chosen to allow the target group to identify with either one of them, serving as relatable “coping models” throughout the different modules.

#### 3.5.4 Content modules

Four different thematic modules have been developed. All modules follow a similar structure. They commence with an animation that explains why the specific factor affects weight and outlines the content of the module. Patient commitment to following the module is then requested. The modules include exercises that target various determinants. It begins with raising awareness and knowledge, for instance, through completion exercises where patients indicate their own challenges related to the theme. Next, various strategies for coping are introduced, with a tailored approach allowing patients to specify areas they wish to work on. Skills are then enhanced through techniques such as implementation intentions/goal setting. Videos featuring individuals with lived experiences (patients who previously have undergone BMS and experienced weight recurrence) provide guidance on how they approach the topic, while potential barriers to sustaining progress are identified and addressed. The module concludes with a milestone and compliment.

#### 3.5.5 Diaries

To raise awareness and facilitate behaviour monitoring, personalized feedback, and goal setting, several diaries have been developed. A food diary allows patients to record their food and beverage intake, timestamps, quantities, notable details (such as emotions), and optionally, a photo of the eating moment. A weight diary enables tracking of weight changes, while a goals diary allows patients to document their goals, mark them as achieved, and set new goals using implementation intentions.

### 3.6 Step 5: Adoption and implementation

In the fifth step a detailed implementation plan was developed**.** To facilitate adoption and implementation, all stakeholders, including HCPs, planners, care coordinators, and NOK location managers were considered. Stakeholders across all organizational levels, including management, were considered to ensure that the intervention is both contextually appropriate and practically feasible, allowing the identification of site-specific needs and barriers to supports tailored implementation planning. For implementation, the management team decided to conduct an evaluation study with the BOT 2.0 at two selected locations. The following implementation strategies have been chosen to facilitate implementation: To raise awareness among implementers, newsletters were distributed among all employees, and presentations on the progress of intervention development and implementation were given in meetings at pilot locations. Education material, e-learnings, two training sessions and instruction materials for the online work environment were developed. We collaborated with managers and planning coordinators and developed new work processes to incorporate the BOT 2.0 into the treatment program.

### 3.7 Step 6: Evaluation planning

Through the establishment of evaluation criteria, the intervention can be assessed against predetermined objectives. Based on the results, further adjustments can be made, and if necessary, the intervention can be scaled up or replicated. We intend to evaluate the intervention in a pilot study using Saunders' framework in the following key areas: reach, dose delivered/received, satisfaction, and fidelity [54]. A mixed methods design will be used, combining questionnaires, interviews, platform data, and electronic patient files. A comprehensive evaluation plan will be detailed in a subsequent publication.

## 4 Discussion

The aim of this study was to describe the systematic further development of the BOT 2.0 intervention for weight recurrence after MBS. The systematic development, guided by IM, resulted in a blended-care intervention that addresses relevant determinants of health behaviour using evidence-based methods. The IM protocol ensured the inclusion of the target group and implementers as well as important evidence-based elements in the intervention.

### 4.1 Limitations and challenges

Severe obesity is a chronic, relapsing disease characterized by a complex interplay of a large variety of contributing factors. It was not feasible to address all relevant factors within the scope of the intervention. Consequently, the systematically developed intervention focused selectively on those causes deemed most important and feasible based on prior research by the planning group within the clinical setting. Whether this is sufficient to prevent weight recurrence and improve patient well-being, will be assessed through ongoing evaluations. Given the complexity of obesity, It is possible that additional factors, beyond the scope of a clinical intervention, such as environmental influences, may be crucial for achieving the desired outcomes. Moreover, for a subset of patients, lifestyle optimization alone may be insufficient, necessitating consideration of surgical revision or pharmacological treatment.

During the real-world development and implementation planning, of the BOT 2.0 intervention, several barriers and challenges emerged. The research process involved balancing scientific rigor with the limitations of practical application in terms of finance, ICT, and agreements with health insurers. For instance, the needs assessment clearly indicated that patients desired additional live peer support or sessions with experts who had lived experience, but these elements could not be integrated into the clinic's program. Also, ideas for more automated tailoring, automatic feedback, and an extensive library of relevant information in the app were proposed, but these features did not align with the app format provided by our developer. IM acknowledges that structural barriers are common during the transition from design to practical implementation and encourages iterative adaptation, feasibility checking, and collaborative decision-making with stakeholders. In line with this, we co-created alternative solutions, such as the incorporation of videos featuring role models with lived experiences and encouraging patients to save app pages containing relevant information to their favourites, thereby creating a personalized library. Therefore, despite these challenges, a systematically developed intervention was successfully established.

This study also has several strengths. When examining the key elements of effective eHealth interventions, they are well-represented in the BOT 2.0 including goal setting, self-monitoring, counsellor feedback and communication, shaping knowledge, and the use of a structured program, combined with personalized contact [55,56]. In addition, the target population was involved in all steps of the protocol, which hopefully enhances engagement with the intervention.

### 4.2 Innovation

This study demonstrates the novel application of the Intervention Mapping (IM) protocol to the development of a blended-care intervention for patients experiencing weight recurrence following MBS. The systematic approach led to the creation of an intervention that integrates in-person support with tailored eHealth components, addressing key patient and healthcare professional needs identified in the needs assessments. This development process provides a transferable framework that can inform the design of similar interventions in other clinical contexts and international settings. Future research will evaluate the effectiveness of the intervention in achieving its intended outcomes, with results to be reported in forthcoming publications.

### 4.3 Conclusion

The IM protocol proved valuable during the development of the BOT intervention. It demonstrated the feasibility of actively involving both the target population and implementers. In the resulting intervention, relevant determinants are assessed, allowing patients to determine which specific factors they wish to address. With this approach the intervention targets the personal and relevant determinants of weight recurrence.

## CRediT authorship contribution statement

**Vera Voorwinde:** Writing – review & editing, Writing – original draft, Project administration, Methodology, Investigation, Conceptualization. **Ingrid H.M. Steenhuis:** Writing – review & editing, Supervision, Conceptualization. **Ignace M.C. Janssen:** Writing – review & editing, Supervision, Conceptualization. **Valerie M. Monpellier:** Writing – review & editing, Supervision, Conceptualization. **Maartje M. van Stralen:** Writing – review & editing, Supervision, Methodology, Investigation, Conceptualization.

## Source of funding

This manuscript is not funded by any specific grant from funding agencies in the public, commercial, or not-for-profit sectors.

## Declaration of competing interest

The authors declare the following financial interests/personal relationships which may be considered as potential competing interests: V. Voorwinde and V.M. Monpellier are employed by the Dutch Obesity Clinic (Nederlandse Obesitas Kliniek). I.M.C. Janssen is the former medical director and consultant for the Dutch Obesity Clinic.

## Appendix A Supplementary data

Tables of Methods and Applications. Image 1
