# Supplementary material for: Getting back on track: Development of a blended-care intervention for weight recurrence after metabolic bariatric surgery using intervention mapping
Source: PEC Innov. 2025 May 18;6:100404. doi: 10.1016/j.pecinn.2025.100404 (PMC12148715; doi:10.1016/j.pecinn.2025.100404)
Supplement: Supplementary file 1 — Tables of Methods and Applications. [file mmc1.docx]

| **Behavioral Outcome** | **Determinants and**  **Change objectives** | **Method** | **Application** |
| --- | --- | --- | --- |
| Choose to actively engage in the Back on Track program | Knowledge about weight recurrence and its causes | Consciousness raising  Information  Early commitment | Information about the BoT  Animation of the visual communication board and potential content of the BoT  Answer question about wanting to start to take action |
|  | Normative beliefs about weight recurrence  Outcome expectations | Modeling  Verbal persuasion | Peer video with a coping model who demonstrates that he/she got back on track with professional help |
|  | Knowledge of individual causes and opportunities to get back on track | Feedback | Online Back on Track questionnaire with outcome per domain |
|  | Outcome expectations | Motivational Interviewing  Tailoring  Goal setting | Determine focus in counseling session,  Personal recommendation for online module  State goal for the BoT in the app |
|  | Self-efficacy | Reinforcement | Praise and reinforcement by HCP and in the app |

| **Behavioral outcome:** | **Determinants and Change objectives** | **Method** | **Application** |
| --- | --- | --- | --- |
| Manage stress in a healthy way | Knowledge of stress and its consequences (on weight) | Information  Elaboration | Animation and infographic about stress  Excercise with ticking boxes and fillong in personal consequences of stress |
|  | Skills  Recognizing a stressor and stress signals | Active learning | Tick boxes and list personal experienced stress signals  List stressors |
|  | Outcome expectations regarding stress management | Modeling  Verbal persuasion | Coping model who has been through stressful times and experienced relapse who had now learned to use adequate coping strategies |
|  | Outcome expectations State that stress can be relieved by deploying adequate coping strategies | Framing | Information on the benefits of stress relieve |
|  | Skills  Selecting and utilizing adequate coping strategies | Planning coping responses | Exercise to differentiate emotional from goal directed coping and make a coping plan |
|  | Normative beliefs  State that it is ok to show emotions and to take time for yourself | modeling | Peer video on prioritizing yourself |
|  | Self-efficacy  Express confidence in ability to cope with stress | Guided practice  Feedback | Practicing, repeating, personal feedback from HCP |
|  | Anticipate perceived barriers such as the tendency to ignore stress | Planning coping responses | Fill in coping plan in app |
|  | Autonomy | Tailoring | Allow patients to choose between a module on relaxation or time for yourself |
|  | Outcome expectations and knowledge  relaxation | Arguments  Information | Information on why relaxation is important: rest and digest |
|  | Knowledge of healthy strategies to relax | elaboration | List examples of personal strategies to relax |
|  | Skills, demonstrate ability to relax | Guided practice | Guided relaxation audio exercise |
|  | Self-efficacy | Review outcome behaviour  Providing reward | Elaborate on the guided relaxation exercise.  Praise/motivational text |
|  | Knowledge. State that a good balance between shoulds and wants is important for lifestyle balance and therefore works protective of relapse | Information | Inforgraphic about lifestyle (im)balance |
|  | Skills,  The ability to prioritize yourself | Implementation intentions  Resistance of social pressure | Filling in if.. then plans, and a coping plan for situations with social pressure |
|  | Normative beliefs  State that significant others approve that I plan time for myself | Shifting perspective | Question what they would advise their best friend |
|  | Self-efficacy | Reinforcement  feedback | Personal feedback during counseling session or in the app |

| **Behavioral outcome** | **Determinants and Change objectives** | **Method** | **Application** |
| --- | --- | --- | --- |
| Follow nutritional guidelines for healthy eating after BMS | Knowledge of the nutritional guidelines | advance organizers  Imagery | Schematic and visual representation of the guidelines |
|  | Skills  Evaluating intake | Monitoring  Feedback | Keep track of food intake in diary  Personal feedback by HCP |
|  | Outcome expectations for improving eating behaviors | modeling | Patient who followed the back on track speaks about eating behaviors |
|  | Autonomy | Tailoring  Motivational interviewing | Allow patient to choose eating behavior to work on  Engage, focus, evoke and plan in counseling session |
|  | Food literacy for  different eating behaviors:  Comparing products  Separate eating and drinking  Healthy snacking  Eating environment  Sufficient time for meals  Eating techniques  Portion control | Information  Guided practice  Implementation intentions | For every skill there is some background information followed by tips to improve this behavior and making guided implementation intentions |
|  | Self efficacy | Providing contingent reward  Reinforcement | Milestone/motivational text  Personal feedback by HCP |

| **Behavioral Outcome** | **Determinants and Change Objectives** | **Method** | **Application** |
| --- | --- | --- | --- |
| Plan meals and important activities | Outcome expectations of planning and goal setting | Modeling | Peer video showing how planning helped getting lifestyle back on track |
|  | Skills and self-efficacy for SMART goalsetting | Guided practice  Chunking  Provide contingent reward  Feedback | Developing and implementing a SMART goal through a step by step example, storing it in the app.  Milestone (motivational text in app)  Feedback in counseling session |
|  | Autonomy | Tailoring  Motivational interviewing | Allowing the patient to choose what to plan (examples: meals, self-care, physical activity).  Engage, focus, evoke, and plan in counseling session |
|  | Outcome expectations of meal planning | Arguments | Demonstrating the benefits of planning and maintaining a regular eating pattern after BMS |
|  | Normative beliefs about meal planning | Modeling | Peer video on meal planning |
|  | Knowledge of the nutritional guidelines after BMS | Advance organizers  Imagery | Schematic/visual representation of guidelines  Infographic example food schedule |
|  | Skills and self-efficacy in meal planning | Guided practice  Implementation intentions  Action planning  Providing reward | Developing an if... then plan step by step following an example  Save and monitor the plan in the app  Milestone/motivational text |
|  | Anticipate perceived barriers such as being from home | Modeling  Planning coping responses | Peer video of coping model  Developing an if... then coping plan step by step following an example |
|  | Autonomy | Tailoring  Motivational interviewing | Allow patient to choose what to plan |
|  | Outcome expectations of planning | Argument | Infographic lifestyle (im)balance |
|  | Normative beliefs about planning | Modeling | Peer video about how planning helped to get back on track |
|  | Skills and self-efficacy in planning | Guided practice  Implementation intentions  Modeling | Step by step implementation intention with examples  Coping model who experienced difficulties prioritizing herself but manages to do so now |
|  | Anticipate perceived barriers such as dealing with resistance from social environment | Planning coping responses | Fill in a coping plan |
|  | Social skills | Guided practice | Exercise with different polite ways to say no |
|  | Skills and self-efficacy in maintaining planning | Providing reward  Feedback | Milestone  Personal feedback/reinforcement by HCP |

| **Behavioral outcome:** | **Change objectives and determinants** | **Method** | **Application** |
| --- | --- | --- | --- |
| Prevent relapse or get back on track after relapse. | Knowledge about lapse/relapse, knowing it is normal when changing behaviours, and doesn’t have to be a problem when one manages to quickly get Back on Track | Information  Framing | Information normalizing lapse/relapse |
|  | Outcome expectations. Learning to recognize high risk situations and to utilize a coping strategy contributes to quickly getting back on track | Arguments | App text and picture: you are more likely to quickly get back on track if you utilize a coping strategy |
|  | Skills  Recognizing  relapse | Elaboration | Tick boxes and fill in personal signals of relapse |
|  | Self-efficacy | Modeling | Peer video showing a coping model who is now maintaining a healthy lifestyle |
|  | Skills  Recognizing personal high-risk situations | Elaboration  Guided practice | Tick boxes and fill in personal high-risk situations |
|  | Coping skills | Guided practice  Planning coping responses | ‘Traffic light’ practice in which high risk situations, signals, and coping responses are filled in. Examples are filled in to guide patients |
|  | Self efficacy | Providing reward  Reinforcement | Milestone, motivational text  Personal feedback |
|  | Outcome expectations of monitoring | Information | Information about the importance of monitoring behaviors in relapse prevention |
|  | Skills  Monitoring | Guided practice  Self-monitoring of behavior | Example diary  Activate app fooddiary  Activate graph to monitor weight |
|  | Self-efficacy | Providing reward  Feedback | Praise/motivational tekst  Feedback from HCP |
